# Predicting Progression of COVID-19 Infection to Prioritize Medical Resource Allocation: A Novel Triage Model Based on Patient Characteristics and Symptoms at Presentation

**DOI:** 10.3389/fpubh.2021.610280

**Published:** 2021-05-11

**Authors:** Yuexing Tu, Xianlong Zhou, Lina Shao, Jiayin Zheng, Jiafeng Wang, Yixin Wang, Weiwei Tong, Mingshan Wang, Jia Wu, Junpeng Zhu, Rong Yan, Yemin Ji, Legao Chen, Di Zhu, Huafang Wang, Sheng Chen, Renyang Liu, Jingyang Lin, Jun Zhang, Haijun Huang, Yan Zhao, Minghua Ge

**Affiliations:** ^1^Zhejiang Provincial People's Hospital, Hangzhou, China; ^2^Zhongnan Hospital, Wuhan University, Wuhan, China; ^3^Fred Hutchinson Cancer Research Center, Seattle, WA, United States; ^4^Gennlife (Beijing) Technology Co. Ltd., Beijing, China

**Keywords:** COVID-19, pandemic, risk factor, nomogram, triage

## Abstract

**Background:** The COVID-19 global pandemic has posed unprecedented challenges to health care systems all over the world. The speed of the viral spread results in a tsunami of patients, which begs for a reliable screening tool using readily available data to predict disease progression.

**Methods:** Multicenter retrospective cohort study was performed to develop and validate a triage model. Patient demographic and non-laboratory clinical data were recorded. Using only the data from Zhongnan Hospital, step-wise multivariable logistic regression was performed, and a prognostic nomogram was constructed based on the independent variables identifies. The discrimination and calibration of the model were validated. External independent validation was performed to further address the utility of this model using data from Jinyintan Hospital.

**Results:** A total of 716 confirmed COVID-19 cases from Zhongnan Hospital were included for model construction. Men, increased age, fever, hypertension, cardio-cerebrovascular disease, dyspnea, cough, and myalgia are independent risk factors for disease progression. External independent validation was carried out in a cohort with 201 cases from Jinyintan Hospital. The area under the curve (AUC) was 0.787 (95% confidence interval [CI]: 0.747–0.827) in the training group and 0.704 (95% CI: 0.632–0.777) in the validation group.

**Conclusions:** We developed a novel triage model based on basic and clinical data. Our model could be used as a pragmatic screening aid to allow for cost efficient screening to be carried out such as over the phone, which may reduce disease propagation through limiting unnecessary contact. This may help allocation of limited medical resources.

## Introduction

In December 2019, Chinese and World Health Organization (WHO) health experts identified a growing number of pneumonia of unknown cause cases leading to substantial health issues for many citizens located in Wuhan, China (1, 2). Identified as a virus, infection caused severe respiratory syndromes and commonly used treatments were often ineffective. Today, we now know the cause to be a novel coronavirus known as the severe acute respiratory syndrome coronavirus 2 (SARS-CoV-2), or COVID-19.

COVID-19 is the latest threat to global health. On March 11, 2020, WHO declared COVID-19 to be a global pandemic, as infection cases were reported in at least 114 countries (3). As of March 15, 2021, a total of more than 119,452,269 cases and 2,647,662 deaths were confirmed worldwide and the number of new cases was expected to increase (4). The high number of COVID-19 cases has overwhelmed health systems globally.

As health system resources are limited—even in highly developed countries—it is crucial to conduct clinical research to determine the best utilization of resources. For example, due to the limited number of medical professionals and equipment (e.g., N95 masks and respirators) available at such short notice, it is almost impossible to provide meticulous, high resource (e.g., intensive care level) health care for every single case of COVID-19 infection. However, this may be acceptable given the fact that initial epidemiologic studies demonstrated that most of COVID-19 cases were classified as mild (81% with non-pneumonia or mild pneumonia) and did not require intensive medical care (5). However, this important finding can only help if we can prioritize resources to those who need it most. Thus, it is important for front-line medical professionals to have a reliable and technically easy way to differentiate those at higher risk for severe and critical symptoms from those at lower risk.

Given that health care systems globally are overwhelmed with the exponential growth of COVID-19 cases and health care resources are limited, it is of extraordinary importance to allocate medical resources effectively and fairly (6). Decisions on resource allocation must be able to be made right at initial patient presentation in order to optimize resource use up front. Thus, it is of notable value to develop a triage model using only patient characteristics and clinical data (i.e., data readily available to medical professionals without the need for additional resources, such as laboratory, and/or imaging technology). In the present study, we create and validate such a novel triage model based on patient data from Chinese COVID-19 epicenter.

## Methods

### Data Sources

This retrospective cohort study was approved by Research Ethics Commission of Zhongnan Hospital of Wuhan University (2020032), Jinyintan Hospital (KY-2020-50.01), and Zhejiang Provincial People's Hospital (2020QT068). The requirement of informed consent was waived due to its retrospective design. On March 10, 2020, all medical records of inpatients diagnosed with COVID-19 in Zhongnan Hospital of Wuhan University and Jinyintan Hospital, Wuhan, China were reviewed. Epidemiological, demographic, clinical symptoms, signs, and comorbidities information were extracted from electronic medical records. All data were examined by two of the listed authors (LNS and ZJ) independently to ensure accuracy.

### Definitions

For this study, the severity of COVID-19 infection was defined according to the World Health Organization (WHO) interim guidance (7). Mild type infection is defined as cases where patients have non-pneumonia or mild pneumonia (5). Severe type infection is diagnosed when at least one of the following three diagnostic criteria is met: (1) respiratory distress (RR ≥ 30/min); (2) resting blood oxygen saturation = < 93%; or (3) arterial blood oxygen partial pressure (PaO2)/FiO2 = <300 mmHg. Critical type is diagnosed when at least one of the following three diagnostic criteria is met: (1) respiratory failure needing mechanical oxygenation; (2) shock; or (3) development of other organ failure, requiring intensive care unit (ICU) care. Fever was defined as axillary temperature of at least 37.3°C. Using these criteria, patient cases were divided into two groups: (1) mild, which could be treated via isolation at home or at the temporary hospital; and (2) severe/critical, which should be admitted for inpatient care within a hospital with full resources/equipment as soon as possible.

### Statistical Analysis

Baseline characteristics of the patients of two groups (mild and severe/critical) were described using counts and percentages for categorical variables and medians with interquartile ranges (IQR) for continuous variables. Differences between groups were tested using the χ^2^ test or Fisher's exact test for categorical variables and *t* test or Wilcoxon test for continuous variables, depending on the nature of the distribution. No imputation was made for missing data. To explore factors associated with severe/critical COVID-19 infection, univariate and multivariate logistic regression models were performed. Independent variables with *p* < 0.05 in univariate analyses were entered in a multivariate model, in which the predictors with *p* < 0.05 were further selected in the final multivariate logistic regression model. Sex and age were selected for all multivariate models for effect-adjustment purpose.

A nomogram was developed based on the final model. Internal and independent validations were implemented to evaluate the predictive performance of the derived nomogram, in terms of discrimination and calibration. For internal validation, we used bootstrap resampling with 1,000 samples to compute bias-corrected estimates. For independent validation, to account for potential discrepancy between the model-development dataset and independent-validation dataset, calibration plots were created for the original and recalibrated nomogram, with recalibration based on the intercept and slope framework as originally proposed by D.R. COX (8). Discrimination was assessed by the receiver operating characteristic (ROC) curve and area under the curve (AUC). Calibration was assessed by comparing observed severe/critical COVID-19 rates with predictions from the final model. All statistical analyses were conducted by one of the authors listed as (JYZ), who works as an independent statistician and was not involved in patient care. All statistical analyses were performed using R software, version 3.6.2 (R Foundation for Statistical Computing), and a two-sided α of <0.05 was considered statistically significant for all tests.

## Results

### Demographic Data and Symptoms

A total of 1,181 patients with suspected or confirmed COVID-19 infection were admitted to Zhongnan Hospital of Wuhan University between December 30, 2019 and March 10, 2020. Among these patients, 406 patients (34%) were excluded because they were assumed positive for COVID-19 infection based only on clinical symptoms and/or CT scan prior to testing kits becoming readily available (performed in Hubei Province only in certain period of time). Another 59 cases (5%) were excluded because of missing clinical data in electronic medical records. Ultimately, a final sample of 716 patients (61%) with confirmed COVID-19 infection was included as a training data set. Same set of data of a cohort of 201 patients with COVID-19 from Jinyintan Hospital was included for independent validation.

The basic epidemiological, demographic, clinical characteristics for the training set (medium age 55, 46.9% male) and the validation set (medium age 63, 49.3% male) were shown in Table 1. In the training set, 161 cases (22.5%) were diagnosed as severe/critical type, while in the validation set, 90 cases (44.8%) were diagnosed as severe/critical. In both set, patients in severe/critical group were significantly older than those in mild group (*p* < 0.001). Hypertension, diabetes and cardio-cerebrovascular disease were the most common comorbidities (Table 1). The most common symptoms on admission were fever, cough, dyspnea, and myalgia (Table 1).

**Table 1:** Characteristics of patients in the training and validation cohorts.

	**Training Cohort (Zhongnan Hospital)**				**Validation Cohort (Jinyintan Hospital)**			
	**Mild (*N* = 555)**	**Severe/critical (*N* = 161)**	**Total (*N* = 716)**	***p***	**Mild (*N* = 111)**	**Severe/critical (*N* = 90)**	**Total (*N* = 201)**	***p***
**Sex**				0.003				0.002
Female	311 (56.0%)	69 (42.9%)	380 (53.1%)		67 (60.4%)	35 (38.9%)	102 (50.7%)	
Male	244 (44.0%)	92 (57.1%)	336 (46.9%)		44 (39.6%)	55 (61.1%)	99 (49.3%)	
**Age**				<0.001				<0.001
N	555	161	716		111	90	201	
Median (IQR)	52 (38–62)	62 (54–72)	55 (41–65)		61 (50–68)	67 (58–73)	63 (53–70)	
**Medical professionals**				0.072				NA
No	325 (89.5%)	94 (95.9%)	419 (90.9%)		NA	NA	NA	
Yes	38 (10.5%)	4 (4.1%)	42 (9.1%)		NA	NA	NA	
**Symptom to admission (Days)**				0.933				0.001
N	547	158	705		111	90	201	
Median (IQR)	7 (3–15)	7 (3–15)	7 (3–15)		8 (4–13)	12 (7–16)	10 (5–15)	
**Fever**				0.006				0.275
No	195 (35.1%)	38 (23.6%)	233 (32.5%)		24 (21.6%)	14 (15.6%)	38 (18.9%)	
Yes	360 (64.9%)	123 (76.4%)	483 (67.5%)		87 (78.4%)	76 (84.4%)	163 (81.1%)	
**Weight**				0.489				NA
N	402	120	522		NA	NA	NA	
Median (IQR)	65 (57.5–73.0)	65 (60.0–74.0)	65 (58.0–73.0)		NA	NA	NA	
**Height**				0.64				NA
N	343	120	463		NA	NA	NA	
Median (IQR)	165 (160–170)	165 (160–170)	165 (160–170)		NA	NA	NA	
**BMI**				0.118				NA
N	342	118	460		NA	NA	NA	
Median (IQR)	23.5 (21.5–25.6)	24.1 (22.4–26.0)	23.7 (21.7–25.7)		NA	NA	NA	
**Current smoker**				<0.001				0.177
No	519 (93.9%)	132 (83.0%)	651 (91.4%)		108 (97.3%)	84 (93.3%)	192 (95.5%)	
Yes	34 (6.1%)	27 (17.0%)	61 (8.6%)		3 (2.7%)	6 (6.7%)	9 (4.5%)	
**Former Smoker**				0.002				NA
No	538 (97.3%)	146 (91.8%)	684 (96.1%)		NA	NA	NA	
Yes	15 (2.7%)	13 (8.2%)	28 (3.9%)		NA	NA	NA	
**Alcohol consumption**				0.018				0.054
No	493 (88.8%)	130 (81.8%)	623 (87.3%)		110 (99.1%)	85 (94.4%)	195 (97.0%)	
Yes	62 (11.2%)	29 (18.2%)	91 (12.7%)		1 (0.9%)	5 (5.6%)	6 (3.0%)	
**Hypertension**				<0.001				0.017
No	451 (81.4%)	91 (56.9%)	542 (75.9%)		79 (71.8%)	50 (55.6%)	129 (64.5%)	
Yes	103 (18.6%)	69 (43.1%)	172 (24.1%)		31 (28.2%)	40 (44.4%)	71 (35.5%)	
**DM**				0.016				0.156
No	509 (91.9%)	136 (85.5%)	645 (90.5%)		100 (90.1%)	75 (83.3%)	175 (87.1%)	
Yes	45 (8.1%)	23 (14.5%)	68 (9.5%)		11 (9.9%)	15 (16.7%)	26 (12.9%)	
**COPD**				0.233				0.114
No	542 (98.0%)	152 (96.2%)	694 (97.6%)		111 (100.0%)	88 (97.8%)	199 (99.0%)	
Yes	11 (2.0%)	6 (3.8%)	17 (2.4%)		0 (0.0%)	2 (2.2%)	2 (1.0%)	
**Cardio-Cerebrovascular Disease**				<0.001				0.228
No	535 (96.6%)	125 (78.6%)	660 (92.6%)		102 (91.9%)	78 (86.7%)	180 (89.6%)	
Yes	19 (3.4%)	34 (21.4%)	53 (7.4%)		9 (8.1%)	12 (13.3%)	21 (10.4%)	
**Dyspnea**				<0.001				0.001
No	434 (78.2%)	90 (55.9%)	524 (73.2%)		57 (51.4%)	25 (27.8%)	82 (40.8%)	
Yes	121 (21.8%)	71 (44.1%)	192 (26.8%)		54 (48.6%)	65 (72.2%)	119 (59.2%)	
**Diarrhea**				0.082				0.503
No	519 (93.5%)	144 (89.4%)	663 (92.6%)		106 (95.5%)	84 (93.3%)	190 (94.5%)	
Yes	36 (6.5%)	17 (10.6%)	53 (7.4%)		5 (4.5%)	6 (6.7%)	11 (5.5%)	
**Myalgia**				0.011				0.918
No	499 (89.9%)	133 (82.6%)	632 (88.3%)		104 (93.7%)	84 (93.3%)	188 (93.5%)	
Yes	56 (10.1%)	28 (17.4%)	84 (11.7%)		7 (6.3%)	6 (6.7%)	13 (6.5%)	
**Cough**				<0.001				0.738
No	288 (51.9%)	56 (34.8%)	344 (48.0%)		37 (33.3%)	28 (31.1%)	65 (32.3%)	
Yes	267 (48.1%)	105 (65.2%)	372 (52.0%)		74 (66.7%)	62 (68.9%)	136 (67.7%)	

*BMI, Body Mass Index; DM, Diabetes Mellitus; COPD, Chronic Obstructive Pulmonary Disease*.

### Independent Risk Factor Identification

Univariate analysis identified that sex, age, presence of fever, current smoker, former smoker, alcohol consumption, hypertension, diabetes mellitus, cardio-cerebrovascular disease, dyspnea, cough, and myalgia were significantly associated with progression of COVID-19 from mild to severe/critical (Table 2).

**Table 2:** Risk factors associated with developing severe/critical group COVID-19.

**Variables**	**Univariate**	***P***	**Multivariate**	***P***
	**OR (95%CI)**		**OR (95%CI)**	
Sex (ref: male)	0.588 (0.413–0.839)	0.003	0.696 (0.459–1.057)	0.089
Age (per year)	1.049 (1.036–1.063)	0.000	1.035 (1.019–1.051)	0.000
Medical professionals	0.364 (0.127–1.046)	0.061		
Fever	1.753 (1.171–2.624)	0.006	1.940 (1.204–3.126)	0.006
BMI (kg/m^2^)	1.058 (0.991–1.130)	0.093		
Current smoker	3.122 (1.819–5.359)	0.000	1.894 (1.008–3.559)	0.047
Former smoker	3.194 (1.486–6.862)	0.003		
Alcohol consumption	1.774 (1.096–2.871)	0.020		
Hypertension	3.320 (2.273–4.850)	0.000	1.845 (1.157–2.942)	0.010
DM	1.913 (1.118–3.272)	0.018		
Cardio-cerebrovascular Disease	7.659 (4.228–13.875)	0.000	4.109 (2.086–8.093)	0.000
Dyspnea	2.830 (1.953–4.099)	0.000	2.244 (1.464–3.440)	0.000
Cough	2.022 (1.405–2.912)	0.000	1.723 (1.137–2.611)	0.010
Diarrhea	1.702 (0.929–3.119)	0.085		
Myalgia	1.876 (1.147–3.069)	0.012	1.981 (1.120–3.504)	0.019

*Variables were transformed to their nature logarithms*.

*CI, Confidence Interval; OR, Odds Ratio; DM, Diabetes Mellitus*.

Using the results of the univariate analysis, a multivariate logistic regression model was developed, which identified that sex (man), increased age, presence of fever, current smoker, hypertension, cardio-cerebrovascular disease, dyspnea, cough, and myalgia were independently associated with increased odds of progression of COVID-19 disease from mild to severe/critical. Woman sex was the only characteristic associated with decreased risk of disease progression (Table 2).

### Nomogram Development

The probability of progressing from the mild to severe/critical group was assessed based on the results of the final multivariate logistic regression. The final multivariate logistic regression model for constructing the nomogram can be expressed as $\ln\left(\frac{{\text{P}}_{\text{severe}/\text{critical}}}{1-P_{\text{severe}/\text{critical}}}\right)\;=\;-\;4.91\;+\;0.36\text{ male }+\;0.03\text{ age }+\;0.66\text{ fever }+\;0.64\text{ smoke }+\;0.61\text{ hypertension}$ + 1.41 cardio − cerebrovascular disease + 0.81 dyspnea + 0.54 cough + 0.68 myalgia where P_severe/critical_ denotes the probability for a patient with COVID-19 to progress to severe/critical COVID-19.

### Nomogram Construction and Validation

**Figure 1:**
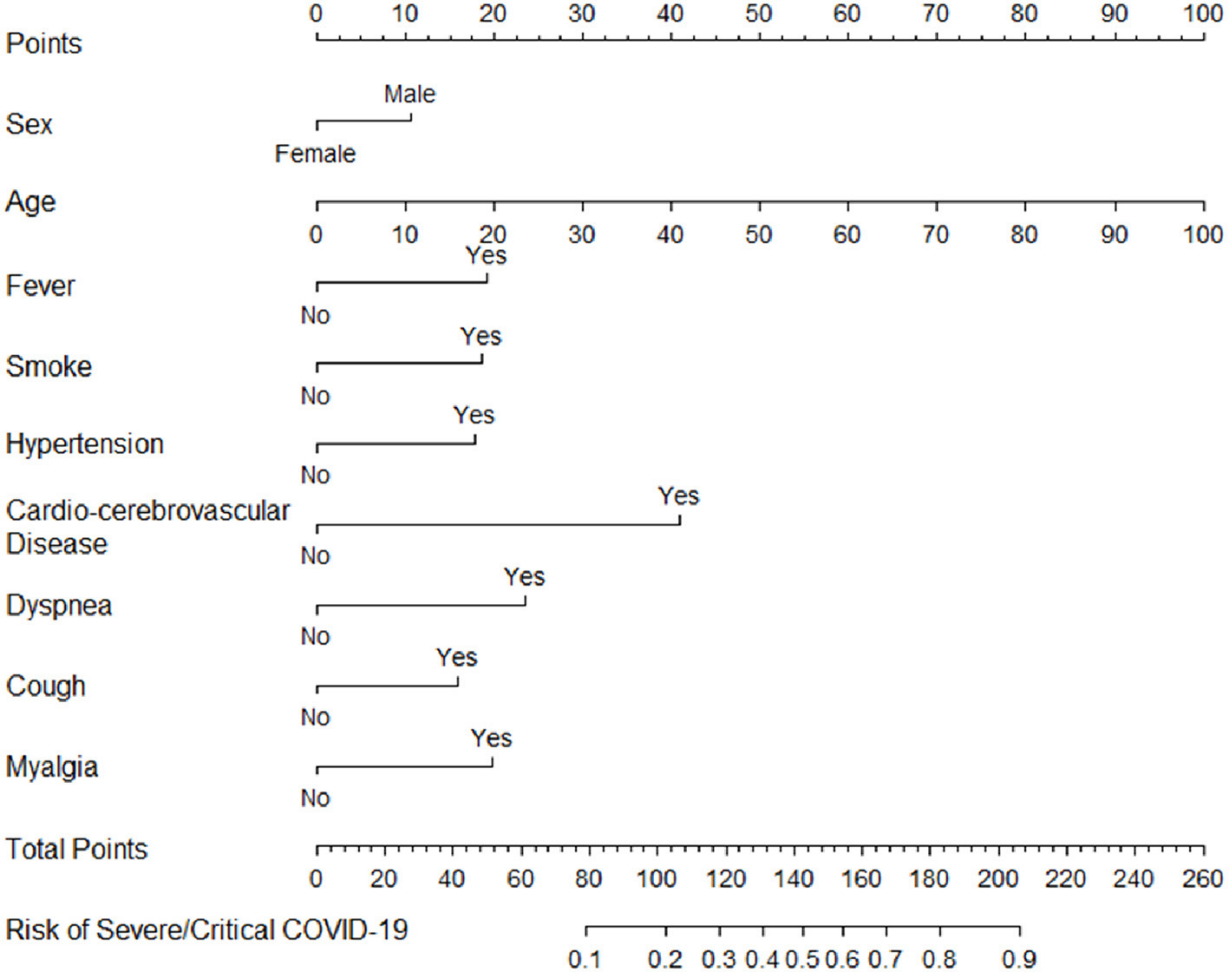
Nomogram of probability to develop severe/critical COVID-19. To use the nomogram, draw an upward vertical line from each covariate to the points bar to calculate the number of points. Based on the sum of the covariate points, draw a downward vertical line from the total points line to calculate the probability of developing severe/critical COVID-19.

A prognostic nomogram for early recognition of those cases that would likely progress to severe/critical cases was constructed using the multivariate logistic regression results. Points were assigned to the identified factors according to the absolute maximum beta value based on the logistic regression model, given that the units are different for the continuous (age) and categorical predictors (sex, fever, smoke, hypertension, cardio-cerebrovascular disease, dyspnea, cough, and myalgia). Though with the smallest beta coefficient of 0.03, the calculated absolute maximum beta value (Beta × value range of the predictor) of age is 0.03 × 89 = 2.67, which means that it has the greatest impact on the probability of the event compared with the other seven predictors (Figure 1). As shown in the nomogram, patients with the following characteristics were more likely to progress to the severe/critical group: sex (man), older in age, presence of fever, current smoker, hypertension, cardio-cerebrovascular disease, dyspnea, cough, and myalgia. Summing all points led to a total score. Locating the total score on the nomogram scale, the risk of progressing to the severe/critical group could be determined at patient presentation.

### Internal and External Independent Validation

**Figure 2:**
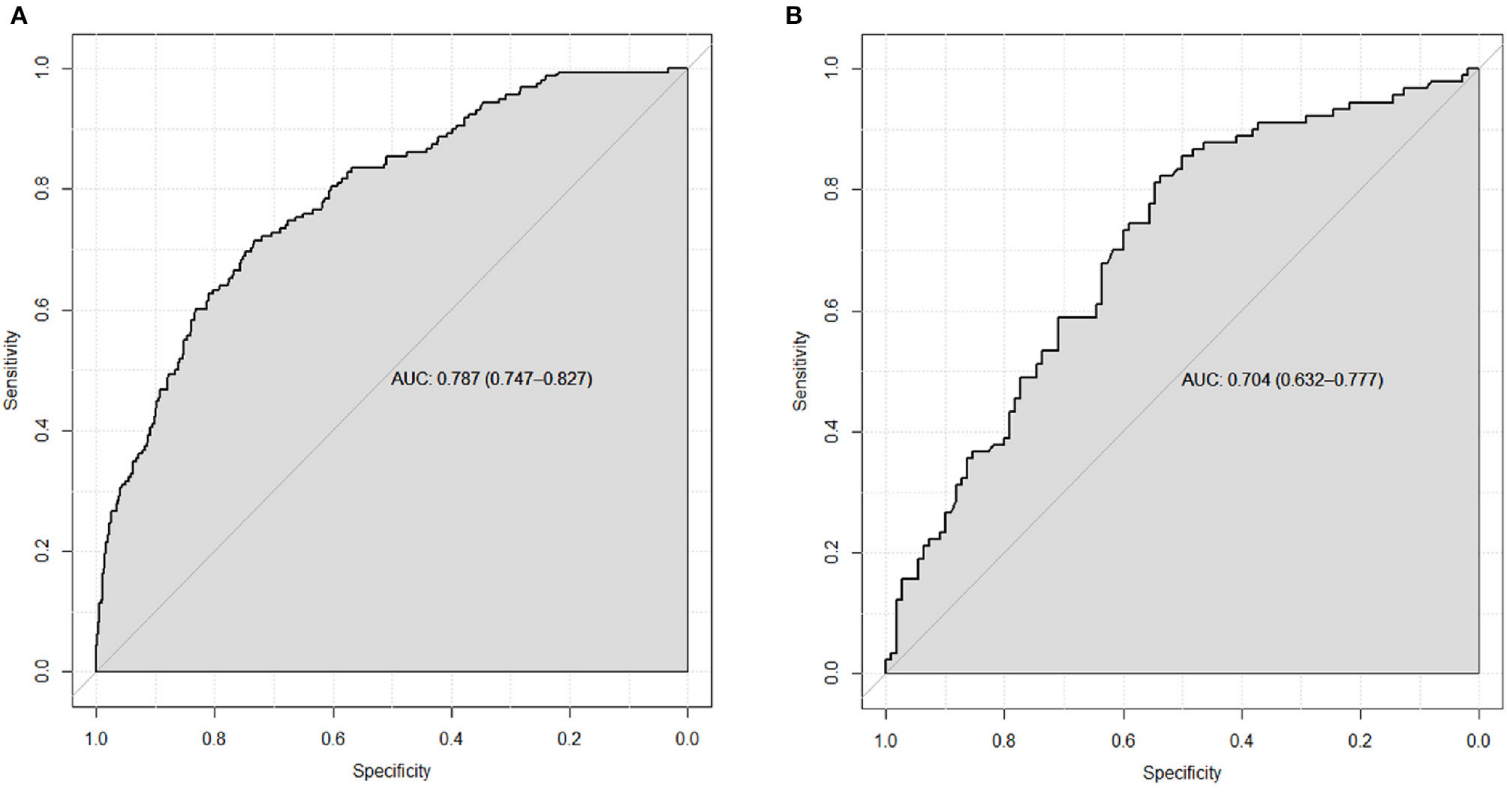
**(A)** ROC curve for the nomogram based on the full Zhongnan Hospital dataset. The bias-corrected AUC is 0.772 based on internal validation using bootstrap resampling (1,000 patients) **(B)** ROC curve from an external, independent validation using the Jinyintan Hospital dataset. The estimate of AUC and its 95% confidence interval are shown in the plots. Key: ROC, receiver operating characteristic. AUC, area under the curve.

**Figure 3:**
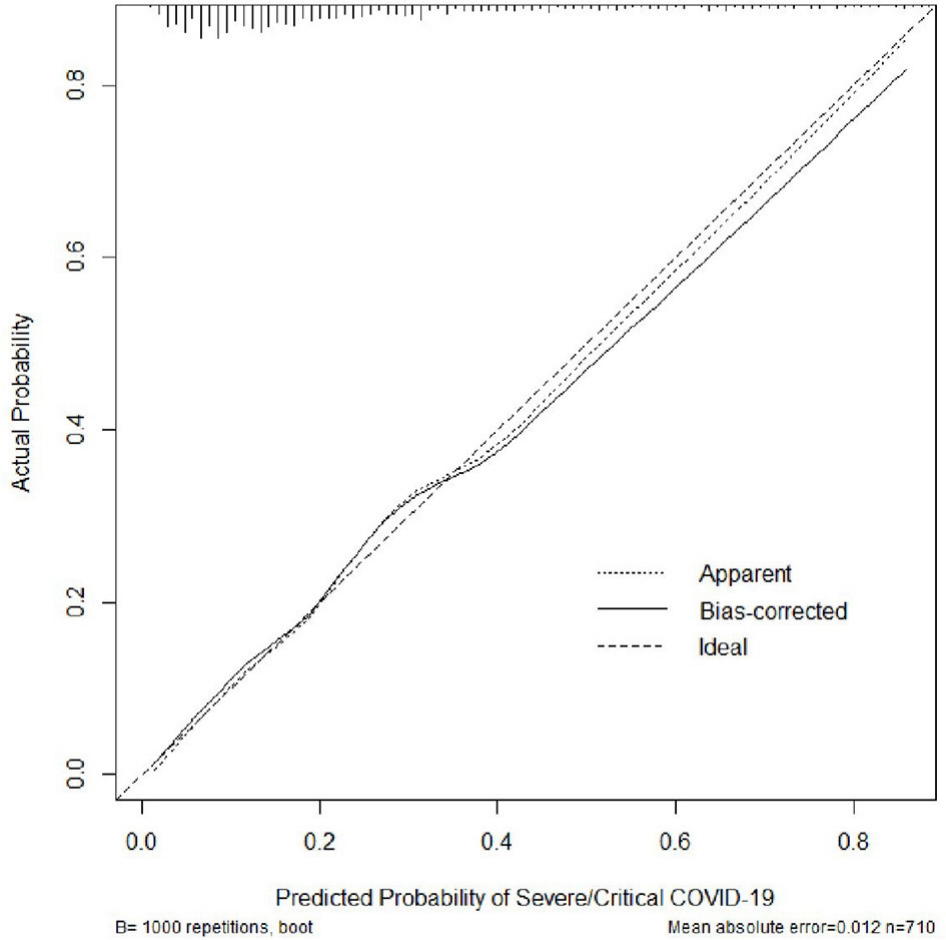
Calibration plot for the nomogram. The bias-corrected (overfitting-corrected) estimates of predicted vs. observed values were obtained based on bootstrap resampling with 1,000 samples for internal validation purpose.

**Figure 4:**
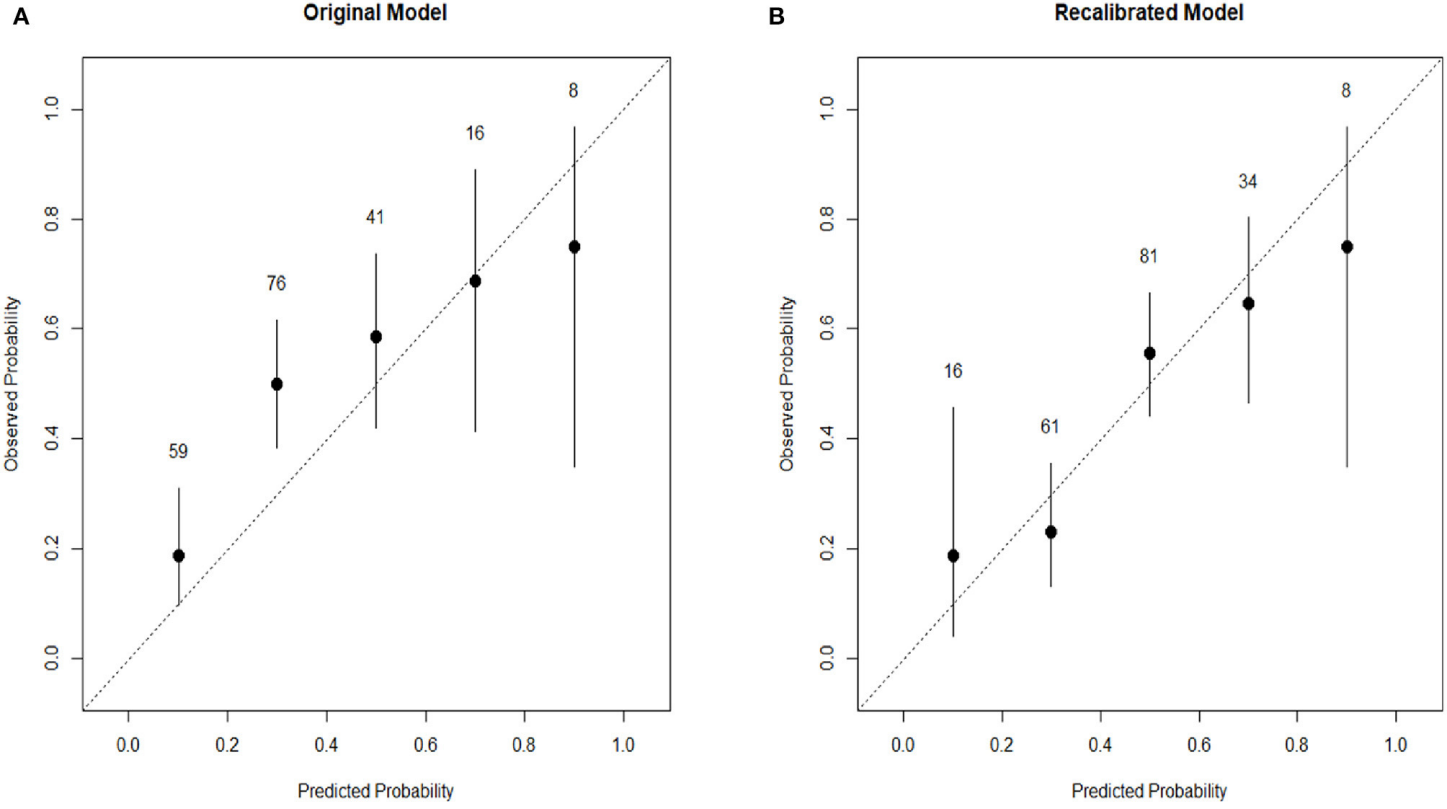
**(A)** External calibration plot for Jinyintan dataset based on the original nomogram. **(B)** External calibration plot for Jinyintan dataset based on the recalibrated nomogram. The nomogram was recalibrated by the intercept and slope framework as originally proposed by D.R. Cox.^8^ The plots are grouped into five bins based on their predicted probabilities, and then the bin prevalence (the ratio of plots in this bin with observed number of severe/critical COVID-19 vs. the total number of plots in this bin) is calculated for each bin. The confidence interval for each bin is also plotted, and the total number of plots is labeled above each the bin. Confidence intervals are calculated for the binomial bin counts using the F distribution.

To evaluate the discrimination of the model and to reduce overfitting bias, internal validation was performed using a bootstrapping technique with 1,000 resamples as qualified. Figure 2 showed the internal validation of the nomogram using a receiver operating characteristic (ROC) curve with an area under the curve (AUC) of 0.787 (95% confidence interval [CI]: 0.747–0.827). We performed external independent validation of our nomogram as well, which demonstrated as AUC of 0.704 (95% CI: 0.632–0.777). The calibration curve showed excellent accordance between the nomogram prediction and the actual observation of severe/critical cases of COVID-19 (Figure 3). An external calibration plot for Jinyintan dataset based on the original nomogram and on the recalibrated nomogram is shown in Figure 4.

## Discussion

The COVID-19 global pandemic has caused great strain on the world's economies and health systems. Without a vaccine or therapeutic available, the number of confirmed cases continues to rise in many areas with many patients requiring hospitalization and a great deal of health care resources. However, health care resources are limited and optimizing their use is critical to successfully tackling this pandemic. In this present study, patient and clinical (non-laboratory) data on 917 patients from two different hospitals, Zhongnan Hospital of Wuhan University and Jinyintan Hospital, in Wuhan, China with confirmed COVID-19 infection were retrospectively reviewed. Step-wise multivariate logistic regression was used to identify risk factors for progression from mild to severe/critical disease. This information was utilized to produce a nomogram predictive model. Men, older in age, presence of fever, current smoker, hypertension, cardio-cerebrovascular disease, dyspnea, cough, and myalgia were all characteristics associated with higher risk for disease progression. Woman sex was the only protective factor. This information can help medical professionals and governments maximize the use of their medical resources by prioritizing patients with greater odds of progressing to severe/critical disease.

Given COVID-19 is a novel coronavirus that was only identified in December 2019, there is an overall paucity of literature to date. However, of the limited prior research, one previous study indicated that older age was an important independent variable associated with mortality in critical COVID-19 patients (9). While our study did not directly examine mortality, our research identified the importance of older age as a variable associated with the progression of COVID-19 disease from mild to severe/critical. The underlying mechanism causing age-related issues could be an age-dependent deficiency in B-cell and T-cell function and the dysfunction of viral elimination due to the excess production of type 2 cytokines, leading to prolonged pro-inflammatory responses (10).

In addition to age, sex is an important factor to consider. The limited literature to date reported that men account for a high proportion of COVID-19 cases, ranging from 58 to 67% (9, 11–13). One hypothesis as to why the literature from China suggests this disease predilection for men is that majority workers in Huanan Seafood Wholesale Market, where the disease appears to have originated, were men (14, 15). Intriguingly, our sample was nearly split evenly by sex. However, our results indicate that men with COVID-19 infection have higher risk for disease progression. While more severe disease in men is consistent with media reports, this is the first study, to our knowledge, that confirms this finding scientifically. However, additional research from other pandemic epicenters is warranted to further evaluate the impact of sex on disease progression and mortality (16).

Presenting clinical symptoms are also crucial elements of initial evaluation of patients with COVID-19 infection. One of the most common presenting symptoms of COVID-19 infection is fever (5, 14, 17). In our study, we found that 67.5% of the cases had fever at presentation. The impact of fever on poor clinical outcomes could be associated with IL-6, which is generally known as a strong pro-inflammatory cytokine and highly expressed in non-survivor groups with severe/critical disease in previous studies (9, 18). The other three symptoms that were independently associated with increased risk of disease progression were dyspnea, cough, and myalgia. Because of the impact of COVID-19 infection on the respiratory system, these were not unexpected finding; however, it remains important for front-line medical professions to consider these specific symptoms as alarming risk factors when treating patients who initially present for care with COVID-19 infection.

Comorbidities are also important to consider when evaluating risk factors for disease progression. Among all of the comorbidities analyzed, hypertension and cardio-cerebrovascular disease were associated with disease progression. Our results showed a significantly higher proportion of patients with hypertension in the severe/critical group than in the mild group (43.1 vs. 18.6%, *p* < 0.01). Hypertension was identified as a risk factor for disease progression, which is partially consistent with previous studies (9, 12, 14). Cardio-cerebrovascular disease was also significantly associated with higher risk of disease progression in our model. Despite its low incidence (7.4% in training cohort and 10.4% in validation cohort), cardio-cerebrovascular disease is of notable concern and medical professionals should be aware of such a diagnosis. Cardio-cerebrovascular disease is a well-known risk factor due to its strong association with all-cause dementia and depression and all-cause mortality (19–21). Our previous study also found that the cases with COVID-19 who was transferred to ICU had a higher proportion of cardio-cerebrovascular disease comorbidity (14).

There are several limitations of our study. First, our analysis included patients from only one country; therefore, the generalizability of our findings to other areas of the world is unknown. However, our findings scientifically verify many of the global media reports and can be considered by public health officials making resource utilization decisions. Second, we included all patients with confirmed COVID-19 infection at their time of initial presentation; however, we did not account for any difference in the duration of symptoms prior to presentation. Because all were aware of this concerning disease, we suspect many did not present with delay. Further, by including all who presented for care, we feel selection biased was reduced. Third, we did not include results from any laboratory or radiographic tests. Such information, could potentially provide additional insight as to factors associated with disease progression. However, our model provides an efficient and easy approach to triaging patients at initial presentation based strictly on patient characteristics, comorbidities, and symptoms. Further, this type of approach is of value in areas where medical supplies and resources are of substantial shortage. Lastly, due to our limited sample size and retrospective cohort study design, we believe a prospective, randomized clinical trial with larger sample size would be helpful to confirm our findings and/or validate new findings. However, given the overwhelming nature of this global pandemic, such a study design may be challenging to perform, especially as new and experimental interventions are being introduced nearly daily. Our study demonstrates the natural disease process for those not undergoing experimental therapeutic intervention.

Overall, we determined which factors are associated with progression of COVID-19 infection from mild to severe/critical. Based on these results, a validated nomogram was developed to help triage patients at presentation and then externally validated. We believe our study findings could be applied in outpatient clinic or emergency department settings to better triage the growing number of newly confirmed COVID-19 cases during this global pandemic. This could help optimize resource utilization within health care systems globally, which is critical at this time of concerned shortages.

## Data Availability Statement

The raw data supporting the conclusions of this article will be made available by the authors, without undue reservation.

## Ethics Statement

This retrospective cohort study was approved by Research Ethics Commission of Zhongnan Hospital of Wuhan University (2020032), Jinyintan Hospital (KY-2020-50.01), and Zhejiang Provincial People's Hospital (2020QT068). The requirement of informed consent was waived due to its retrospective design.

## Author Contributions

YT, XZ, LS, JZhe, JZha, HH, YZ, and MG conceived of the presented idea. JWa, MW, JWu, JZhu, RY, YJ, LC, DZ, HW, SC, RL, and JL collected clinical data for this study. JZhe, YW, and WT performed statistical analysis. JZha, HH, YZ, and MG encouraged YT, XZ, LS, and JZhe to investigate and supervised the findings of this work. All authors discussed the results and contributed to the final manuscript.

## Conflict of Interest

The authors declare that the research was conducted in the absence of any commercial or financial relationships that could be construed as a potential conflict of interest.
